# Phytochemical derivatives targeting fliJ flagellar protein from *Escherichia coli*

**DOI:** 10.6026/97320630014465

**Published:** 2018-10-30

**Authors:** CR Hemalatha, PA Abhinand, Maithreyi Iyer, Benedict C Paul, Anupma Jyoti Kindo, Thyagarajan Ravinder, Dhanapalan P

**Affiliations:** 1 Sri Ramachandra Institute of Higher Education and Research, Porur, Chennai – 600116; 2 Govt. Kilpauk Medical College and Hospital,Chennai – 600010; 3 Madras Veterinary College, Chennai – 600007

**Keywords:** Biofilms, *E. coli*, fliJ, flagellar protein, phytochemical derivatives

## Abstract

Approximately 50 per cent of nosocomial infections are caused by the use of indwelling medical devices. The surfaces of devices are ideal sites of attachment for bacterial cells and an increase in biofilm formation. Biofilms have been a constant concern due to their complex extracellular matrix (ECM) resulting in multiple drug resistance. *E. coli* is known to associate with biofilms. Therefore it is of interest to identify the proteins associated to biofilm formation in Escherichia coli through literature survey, investigate their protein-protein interactions and identify indispensible proteins of biofilm formation. These proteins were further analyzed and fliJ was identified as the target, based on betweenness, centrality and radiality. 87 phytochemicals were found to be associated with the microbe in question and were docked with the target using Molegro Virtual Docker (MVD) 5.0. The results showed that geranyl pyrophosphate, ferulic acid 4-o-b-d-glucuronide, 5-8'-dehydrodiferulic acid and geranyl diphosphate showed maximum activity. A combinatorial library of 96 models was generated using the four phytochemicals binding with fliJ.

## Background

Biofilms have been a constant concern due to their compact yet complex extracellular matrix (ECM). A major concern associated with their eradication is due to their complex signalling and diversity in structural composition 1. This allows microorganisms in biofilms to survive and withstand hostile circumstances like starvation and desiccation, thereby enabling them to cause a broad range of chronic infections. Biofilms are often found on surfaces of medical devices. Around 50% of nosocomial infections are caused due to the use of indwelling medical devices such as cardiac pacemakers, catheters, dentures, lenses, prosthetic valves and joint prostheses 2. The surfaces of such devices are ideal sites of attachment for bacterial cells and a raise in biofilm formation has been noticed in the presence of indwelling medical devices 3.

Microbial colonization begins within 24 hours after insertion of catheters 4. Central-venous catheter-related bloodstream infections (CRBSIs) are one of the principal causes of nosocomial infections coupled with morbidity, mortality and cost. CRBSIs are caused by Escherichia coli, Klebsiella pneumoniae, Staphylococcus aureus, Pseudomonas aeruginosa and Acinetobacter baumanii, out of which, eight per cent was attributed by E. coli 5. Biofilms harbour multiple microorganisms and the communication occurs through a complex signalling process - quorum sensing.

It is of interest to identify proteins associated with biofilm formation in Escherichia coli by literature survey, investigate their protein-protein interactions and identify indispensible proteins of biofilm formation. These proteins will further be analyzed to identify an appropriate target based on betweenness, centrality and radiality. Phytochemicals found to be associated with E. coli will be docked with the target protein and a combinatorial library of the identified phytochemicals will be built to enable synthetic production of the ligand.

## Methodology

### Study of Protein-Protein Interactions

338 E. coli proteins involved in biofilm formation were identified using literature survey. Interactions between the proteins were studied using the STRING 10.0 database. The STRING results were further analysed by using Cytoscape and plug-ins, M-CODE and CENTISCAPE.

### Identification of Drug Targets

In graph theory, a clique is a subset of vertices of an undirected graph such that every two distinct vertices in the clique are adjacent and dense cliques are the sub-networks formed using the plug-in, M-CODE. 11 dense cliques were obtained of which 5 dense cliques had a threshold score above 5 in the M-CODE analysis. The MCODE analysis helped to separate the protein networks based on function. CENTISCAPE analysis was done to identify the subnetwork with the maximum interaction of proteins using betweenness, centrality and radiality properties. Maximum betweeness centrality was observed in the flagellar protein subnetwork amongst three proteins: fliJ, fliP and flgN.

### Protein Modelling

The properties of the proteins fliJ, fliP, flgN such as sequence, sequence length, mass and presence of 3-D structures was studied. A PSI-BLAST was run and a template for fliJ protein was obtained. fliJ has a pivotal task in flagellar assembly as it is involved in chemotactic stimuli. The template chosen to model the protein had 100% identity and 88% query coverage. The template used was Chain A of fliJ protein obtained from Salmonella enterica subspecies. Homology modelling of fliJ was performed using Swiss Model. The model obtained was further analysed using ERRAT2, ProSA and PDBsum to check the quality.

### Identification of Lead Molecules against E. coli:

Phytochemicals showing antimicrobial activity against E. coli were identified and their structures were obtained. The phytochemical molecules which satisfied the ‘Lipinski's Rule of Five’ were chosen. Virtual Screening by Molecular Docking Phytochemicals that satisfied with the Lipinski’s Rule of Five was docked with the protein model of fliJ obtained using Molegro Virtual Docker (MVD) 5.0. MVD 5.0 uses MolDock scoring system and it is based on a hybrid search algorithm, called guided differential evolution. This algorithm combines the technique of differential evolution optimization with a cavity prediction algorithm. The modelled protein structure was loaded on to MVD 5.0 platform for the molecular docking process. The built-in cavity detection algorithm of MVD 5.0 was used to identify the potential binding sites which are also referred to as active sites or cavities.

The search algorithm used was Moldock SE and 10 was the number of runs taken while 2000 was the maximum iterations for a population size of 50 having 100 as the energy threshold. At every step, least 'min' torsions/translations/rotations were sought and the molecule having the lowest energy was preferred. After molecular docking simulation, the poses (binding modes) obtained were classified by re-rank score.

Using the ligand preparation module of MVD 5.0, the selected ligands were manually prepared. Bond order, flexible torsion and the ligands were deducted. After the careful removal of hetero atoms and water molecules, the target protein structures were prepared and its electrostatic surface was produced. The molecular docking was subjected to amino acid residues which were found to be a part of the interaction of fliJ with geranyl phosphates and ferulic acids. The grid resolution was set at 0.3 Å. The maximum interaction and maximum population size were set at 1500 and 50 respectively 6.

A combinatorial library was developed using the phytochemical molecules which showed maximum activity with the target protein, using SmiLib v2.0. 7 SmiLib is a free, platform independent software tool for rapid combinatorial library generation in the SMILES notation.

## Results

### Study of Protein-Protein Interactions

**Figure 1:**
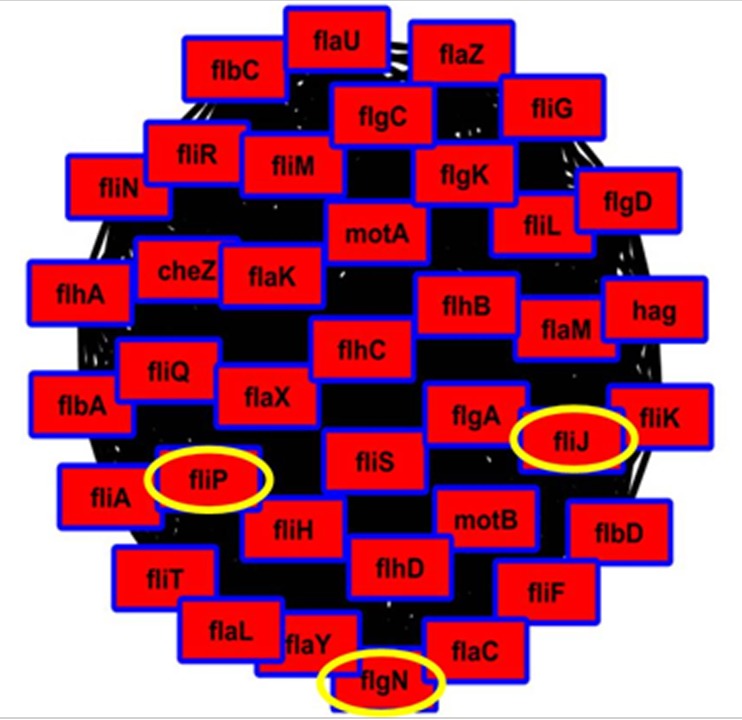
Sub-network showing a score of 36 (threshold: 5) for proteins showing maximum betweenness. The association of fliJ in the network is shown.

The Centiscape Plug-in of Cytoscape is based on the property of maximum betweenness centrality, centrality and radiality. These are graph theory and network analysis terminologies which mean a measure of centrality in a graph based on shortest paths (betweenness centrality), identification of the most important vertices within a graph (centrality - where its applications include identifying the most influential protein in a network) and a measure of the number of nodes reachable from a central node in a network (radiality). Among the interacting proteins in the subnetwork (dense clique) in Cluster 1, three proteins (Figure 1) were selected for further study – flgN, fliP and fliJ.

### Protein Modelling of fliJ:

**Figure 2:**
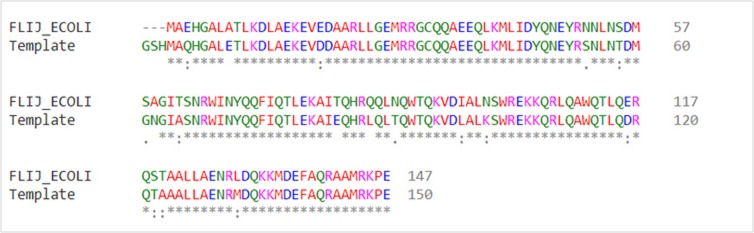
Pair-wise alignment of fliJ protein of Escherichia coli against the template with sequence similarity of 88% from Salmonella typhi; :denotes conserved substitution and denotes semi conserved substitution; *denotes identical and fully conserved

**Figure 3:**
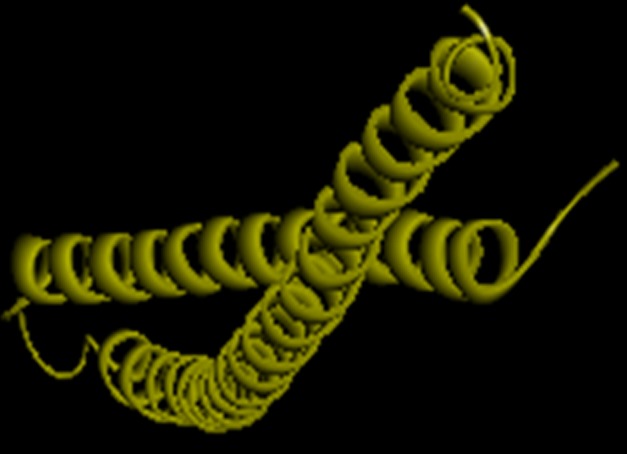
Structrual model of fliJ protein created using Discovery Studio

The properties of the proteins fliJ, flip and flgN, such as the amino acid sequence length, mass and presence of 3-D structures were studied in UniProtKB. A PSI-BLAST alignment (Figure 2) was run and a template for fliJ protein was obtained. The template chosen to model the protein had 100% identity and 88% query coverage. The template used was Chain A of fliJ protein obtained from Salmonella enterica subspecies. Homology modelling of fliJ was performed using Swiss Model. The model obtained was further analysed using ERRAT2, PDBSum and ProSA to check the quality. The ERRAT2 analysis showed that the modelled protein structure showed an overall Quality Factor of 99.2188 which is acclaimed to be a very good score. In PDBSum, Ramachandran plot analysis was done and based on literature, an analysis of 118 structures of resolution of at least 2.0 Angstroms and R-factor no greater than 20.0, a good quality model would be expected to have over 90% in the most favoured regions (A, B, L). 8 The obtained 3-D model (Figure 3) shows 95.4% in the most favoured region showing that the overall quality is good. ProSa analysis shows the energy minimized regions in the modelled protein. Lower the energy of the molecule higher will be its function. It also exhibits the errors in the 3Dmodel. 9

### Identification of Lead Molecules against E. coli:

A total of 87 molecules (Table 1) were found to be having antimicrobial activity against E. coli by literature survey.

### Molecular Docking:

All the 87 phytochemical molecules obtained were docked with the fliJ protein. The molecular docking results were tabulated for all compounds. Of all compounds, out of the many molecular docking poses, only the ones which have the highest moldock score and relatively good hydrogen bond interaction were chosen. The best few compounds which displayed very good affinity with the interaction site were selected.

The molecular docking results (Table 2) showed that four molecules Geranyl Pyrophosphate, Ferulic Acid 4-O-b-DGlucuronide, 5-8'-Dehydrodiferulic acid and Geranyl Diphosphate showed very good molecular docking results based on high molecular docking scores and interacting amino acids. Tryptophan 66 is found in the binding pocket.

## Discussion

**Figure 4:**
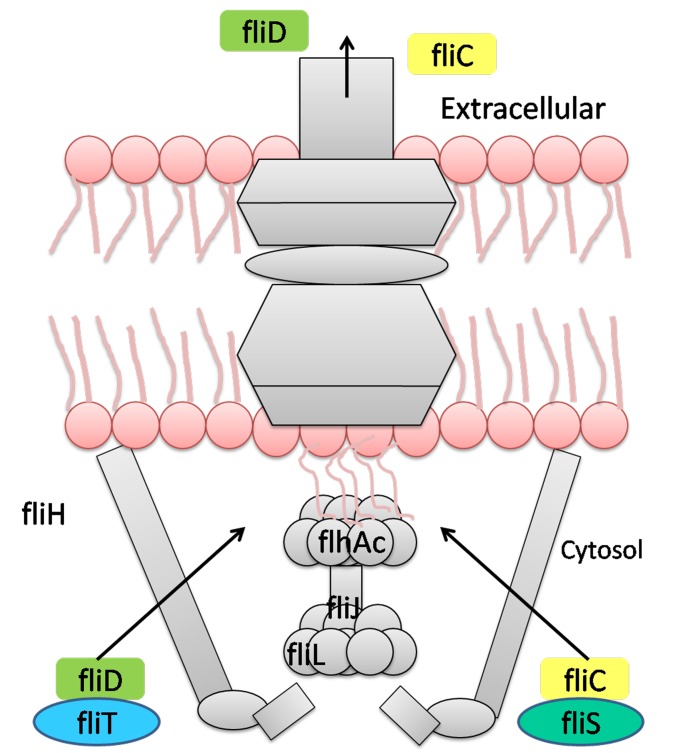
fliJ protein in the flagellar apparatus

Biofilms are bacterial communities which are multi-cellular and sheathed in an extracellular matrix. It is known that biofilms are associated with 80% of all bacterial infections. 10 Antibiotics treatment is often ineffective. It is of interest to identify phytochemicals that target essential proteins in E. coli. 11 fliJ is one of three soluble components of the flagella, (Figure 4) along with fliH and fliI. 12 The fliJ protein takes part in chemotactic events and mutations in fliJ marks the failure to counter chemotactic stimuli. 13They form the ATPase complex and are evolutionarily related to components of the VoV1 and FoF1 rotary ATPases. 14-19The ATPase complex participates in the sorting and entry of substrates into the export gate, while the movement of substrates into the central channel of the flagella is driven by the proton motive force. 20-22

The principle objective of our study was to identify phytochemicals which may target some essential proteins in Escherichia coli. The interacting amino acids of geranyl pyrophosphate were Arg50, Tyr69, Trp66, showing a strong physical interaction between the flagellar protein, fliJ and the phytochemical, geranyl pyrophosphate. The other phytochemicals which showed good activity with the target are ferulic acid 4-o-b-d-glucuronide, 5-8'- dehydrodiferulic acid and geranyl diphosphate. The common interacting amino acid is Trp66, which is the running thread which happens to be in the list of interacting amino acids of all the four phytochemicals which showed maximum activity in MVD 5.0. MCODE analysis was performed in Cytoscape and 11 subnetworks were obtained of which 5 subnetworks had a threshold score above 5. The M-code analysis helped to separate the protein networks based on function. CENTISCAPE analysis was done to identify the subnetwork with the maximum interaction of proteins using betweenness, centrality and radiality properties. Maximum betweeness and centrality was observed in the flagellar protein subnetwork amongst 3 proteins: fliJ, fliP and flgN.

The properties of the proteins fliJ, fliP, flgN such as sequence, sequence length, mass and presence of 3-D structures were studied. A PSI-BLAST was run and a template for fliJ protein was obtained. fliJ plays a role in flagellar assembly as it is involved in chemotactic stimuli. The template chosen to model the protein had 100% identity and 88% query coverage. The template used was Chain A of fliJ protein obtained from Salmonella enterica subspecies. Homology modelling of fliJ was performed using Swiss Model. The model obtained was further analysed using ERRAT2, ProSA and PDBsum to check the quality. A total of 87 molecules were found to be having antimicrobial activity against E. coli by literature survey. All the phytochemical molecules obtained were docked with the fliJ protein. The molecular docking results were tabulated for all compounds. Out of the many molecular docking poses, for every compound, only those with the highest Moldock Score and good hydrogen bond interaction were preferred. A few compounds which showed a very good affinity towards the interaction site were picked.

## Conclusion

Medical biofilms is a ubiquitous threat. Therefore, it is of interest to disrupt biofilms. The molecular interaction between the bacterial flagellar protein fliJ and geranyl pyrophosphate, ferulic acid 4-o-bd- glucuronide, 5-8'-dehydrodiferulic acid and geranyl diphosphate denote probable prevention of biofilm formation in Escherichia coli strains. The phytochemical geranyl pyrophosphate exhibited the highest binding affinity for further consideration against Escherichia coli biofilms.

## Figures and Tables

**Table 1:** Known Phytochemicals used against E. coli

Name of the Phyochemical	Common Name	Pubchem CID
7-hydroxycoumarin (7-HC)	Umbelliferone	5281426
indole-3-carbinol (I3C)	indole-3-carbinol (I3C)	3712
salicylic acid (SA)	salicylic acid (SA)	338
saponin (acer saponin)	Ethyl N-butan-2-yl-N-nitrosocarbamate	275972
saponin	Pregnene Saponin	3010873
Ginkgolic acid	Ginkgolide J	24721483
HNS	HNS 32	3037457
gallic acid	gallic acid	370
ferulic acid 1	Ferulic Acid	445858
ferulic acid 2	Acetylferulic acid	5354677
ferulic acid 3	5-Hydroxyferulic acid	446834
ferulic acid 4	cis-Ferulic acid	1548883
ferulic acid 5	Methyl Ferulate	5357283
ferulic acid 6	Ethyl Ferulate	736681
ferulic acid 7	Ferulic Acid Sulfate	6305574
ferulic acid 8	Ferulic acid 4-glucuronide	6443140
ferulic acid 9	Ferulic Acid-d3	45039253
ferulic acid 10	Ferulamide	6433734
ferulic acid 11	Ferulic Acid Ethylester	65133
ferulic acid 12	2-Hydroxy-3-methoxycinnamic acid	5463156
ferulic acid 13	Phenylethyl-3-methylcaffeate	5284444
ferulic acid 14	trans-p-Coumaric acid 4-glucoside	9840292
ferulic acid 15	Methyl ferulate, (Z)-	10176654
ferulic acid 16	Dihydroferulic acid 4-O-glucuronide	190069
ferulic acid 17	Ferulic Acid-d3 4-O-Sulfate	71316749
ferulic acid 18	Methyl 4-acetoxy-3-methoxycinnamate	5354678
ferulic acid 19	2-Ethylhexyl trans-ferulate	11961066
ferulic acid 20	KSEBMYQBYZTDHS-FIBGUPNXSA-N	57369490
ferulic acid 22	(E)-3-(4-Hydroxy-3-methoxyphenyl)prop-2-enoic acid	71311006
ferulic acid 23	8,8'-Diferulic acid	10475220
ferulic acid 24	IEMIRSXOYFWPFD-BJGSYIFTSA-N	13916049
ferulic acid 25	5-8'-Dehydrodiferulic acid	10385447
ferulic acid 26	Dihydro Ferulic Acid Methyl Ester	126969002
ferulic acid 27	Acetyl Ferulic Acid	69501299
ferulic acid 28	Dihydro-ferulic acid	17865499
ferulic acid 29	5-Hydroxy ferulic acid	54740354
ferulic acid 30	TWSIWBHKRJLZCF-JHZZJYKESA-N	187484
ferulic acid 31	Carbomethoxy-ferulic acid	129663005
ferulic acid 32	Acetyldihydro-ferulic acid	129773815
ferulic acid 33	1-O-Feruloyl-beta-D-glucose	13962928
ferulic acid 34	N-Feruloyl serotonin	5969616
ferulic acid 35	IWKLPOJPPIBQHO-FNORWQNLSA-N	12993148
ferulic acid 36	JWRQVQWBNRGGPK-JZYAIQKZSA-N	53978589
ferulic acid 37	TWSIWBHKRJLZCF-QXOFVJDBSA-N	71316748
ferulic acid 38	(E)-3-(4-Hydroxy-3-methoxyphenyl)prop-2-enoic acid	117064991
flavonoid 1	Ternatin	5459184
flavonoid 2	Eupatin	5317287
flavonoid 3	Laurifolin (Flavonoid)	44257868
flavonoid 4	Eupatoretin	275525
flavonoid 5	Lanceolatin A	6442389
flavonoid 6	Hispidone[Flavonoid]	9997719
flavonoid 7	Genistein	5280961
flavonoid 8	Glabranin	124049
flavonoid 9	Galangin	5281616
terpenoid 1	Carane	79043
terpenoid 2	Cedr-8-ene	521207
DMAPP 1	Dimethylallyl Diphosphate	647
DMAPP 2	Dimethylallyl-PP	15983958
isopentenyl diphosphate 1	Isopentenyl Pyrophosphate	1195
isopentenyl diphosphate 2	3-Methylbut-3-enyl diphosphate	15983957
isopentenyl diphosphate 3	Geraniol Isopentenyl Diphosphate	129761672
DXP	1-Deoxy-D-xylulose 5-phosphate	443201
deoxyxylulose phosphate 1	D-1-Deoxyxylulose-5-P	23420274
deoxyxylulose phosphate 2	Deoxyxylulose Phosphate	129635163
Steroid 1	Testosterone	6013
Steroid 2	Oxymetholone	5281034
Steroid 3	Testosterone Propionate	5995
Steroid 4	M-Dinitrobenzene	7452
Steroid 5	XLRLZPOBHPIDFX-NSHDSACASA-N	6604912
mevalonic acid 1	Mevalonic Acid	439230
mevalonic acid 2	(R)-Mevalonate	5288798
mevalonic acid 3	3,5-Dihydroxy-3-methylpentanoate	4478250
mevalonic acid 4	3,5-Dihydroxy-3-methylpentanoic acid	449
GPP 1	Geranyl Diphosphate	445995
GPP 2	(E)-2-Methylgeranyl diphosphate	51351720
GPP 3	TVKWRLSBRIRPDH-MOHJPFBDSA-N	58934084
GPP 4	UWHNDWYDKGVRGE-UHFFFAOYSA-N	447258
geranyl diphosphate 1	Geranylgeranyl Pyrophosphate	447277
geranyl diphosphate 2	Geranylgeranyl Diphosphate	5497105
farnesol 1	farnesol	445070
farnesol 2	farnesol	1549109
farnesol 3	Farnesyl Pyrophosphate	445713
farnesol 4	Farnesyl Triphosphate	5280571
farnesol 5	Methoxy Farnesol	129724957
MVA	BBYIXLRFQJBTBG-QDSNELGPSA-N	5287406
CDH 2	1,1,2-Trideuterioethene	137677
CDH 3	LQNFEEJOZSCHID-CDWOPPGASA-N	122400891

**Table 2:** Molecular docking results of phytochemicals having maximum interaction with the protein fliJ

S. No.	Phytochemical	Moldock Score	Hbond Energy	Interacting Amino Acids
1	Geranyl Pyrophosphate	-125.216	-13.9796	Arg50, Tyr69, Trp66
2	Ferulic Acid 4-O-b-D-Glucuronide	-117.957	-18.0419	Asp56, Asn54, Ala59, Leu53, Trp66, Gln70
3	5-8'-Dehydrodiferulic acid	-114.263	-7.06335	Trp66, Asn164, Arg65, Thr62
4	Geranyl Diphosphate	-113.892	-17.136	Trp66, Thr62, Ala69, Asp56, Ser63
